# Rethinking Surgical Margins in Colorectal Liver Metastases: Vessel-Skeletonized Parenchymal-Sparing Hepatectomy (VESPAH) and Treatment Continuity

**DOI:** 10.3390/cancers18213492

**Published:** 2026-10-29

**Authors:** Yuzo Umeda, Tomokazu Fuji, Kosei Takagi, Kunitoshi Shigeyasu, Mikiya Shine, Kei Tamura, Munenori Takaoka, Takeshi Nagasaka

**Affiliations:** 1 Department of Hepato-Biliary-Pancreatic and Transplant Surgery, Ehime University Graduate School of Medicine, Toon 791-0295, Japan; 2 Department of Gastroenterological Surgery, Okayama University Graduate School of Medicine, Dentistry and Pharmaceutical Sciences, Okayama 700-8558, Japan; 3 Department of Clinical Oncology, Kawasaki Medical School, Kurashiki 701-0192, Japan

**Keywords:** colorectal liver metastases, surgical margin, R1 vascular margin, parenchymal-sparing hepatectomy, vessel skeletonization, Laennec’s capsule, treatment continuity, repeat hepatectomy, thermal ablation, VESPAH

## Abstract

Complete (R0) resection remains the primary surgical objective for colorectal liver metastases (CRLM), but margin width cannot be interpreted independently of tumor anatomy, biology, and the amount of functional liver sacrificed to obtain it. This focused narrative review re-examines surgical margins from the perspective of parenchymal preservation and longitudinal multimodal treatment. We distinguish parenchymal R1 exposure (R1par), an undesirable transection-surface event consistently associated with local recurrence in retrospective series, from planned vascular R1 detachment (R1vasc), which has shown more favorable outcomes than R1par in selected retrospective series but remains dependent on careful anatomical selection. Vessel-skeletonized parenchymal-sparing hepatectomy (VESPAH) is presented as one operative model for achieving complete macroscopic clearance while preserving intrahepatic vascular architecture; the evidence for this model is confined to retrospective series and is therefore illustrative and hypothesis-generating rather than confirmatory. We further propose “treatment continuity”—preservation of the physiological and anatomical capacity to receive indicated systemic therapy and repeat local treatment—as a reportable surgical outcome. A four-axis framework integrating technical resectability, functional reserve, tumor biology, and future treatment options is outlined, together with a minimum set of reporting elements for margin phenotype and operational definitions for four candidate measures of treatment continuity. R0 resection should remain the goal, but the quality of hepatectomy for CRLM may be more comprehensively assessed by considering both the margin achieved at the index operation and the treatments that remain feasible thereafter.

## Simple Summary

Surgery offers the best chance of long-term survival for selected patients with colorectal liver metastases, and a negative resection margin remains the preferred surgical objective. In patients with tumors abutting major intrahepatic vessels or with extensive bilobar disease; however, obtaining a wide margin may require removal of substantial functional liver parenchyma. Because recurrence remains common, excessive parenchymal loss at the first operation may limit later systemic therapy, repeat hepatectomy, or local ablation. This review examines the distinction between parenchymal R1 exposure and planned vascular detachment, describes vessel-skeletonized parenchymal-sparing hepatectomy as one technical expression of margin-aware surgery, and proposes “treatment continuity” as an additional surgical outcome. The supporting evidence is retrospective and illustrative rather than confirmatory. The aim is not to justify indiscriminate R1 resection, but to balance complete macroscopic clearance with preservation of liver function and future treatment options.

## 1. Introduction

Surgical resection remains the cornerstone of potentially curative treatment for colorectal liver metastases (CRLM). Historically, the oncological quality of hepatectomy was judged largely by the width of the tumor-free margin. Subsequent studies, however, showed that subcentimeter margins can provide acceptable long-term outcomes provided that microscopic tumor exposure is avoided [1,2,3,4]. R0 resection therefore remains the preferred endpoint, with a margin of at least 1 mm generally considered desirable, but the pursuit of additional margin may require disproportionate loss of functional liver in patients with multiple bilobar tumors or lesions abutting major intrahepatic vessels.

This tension has driven a shift from anatomical radicality toward parenchymal preservation. Ultrasonography-guided “radical but conservative” surgery tailored the transection plane to intrahepatic vascular anatomy [5,6], and subsequent series and meta-analyses have shown that parenchymal-sparing hepatectomy (PSH) can achieve oncological outcomes comparable to more extensive resections while preserving the possibility of further liver-directed treatment [7,8,9,10]. This consideration is particularly relevant in CRLM, where hepatic recurrence is common and long-term survival may depend on repeated combinations of systemic therapy, repeat hepatectomy, and local ablation.

PSH has also challenged the binary interpretation of R0 versus R1. Planned detachment of a metastasis from a major intrahepatic vessel, usually termed an R1 vascular margin (R1vasc), is anatomically distinct from parenchymal R1 exposure (R1par). Retrospective studies suggest that R1par carries a greater risk of local failure, whereas selected R1vasc resections may achieve outcomes closer to R0 resection [11,12,13]. The evidence is not uniform: a recent meta-analysis restricted to minimally invasive liver surgery reported a higher local recurrence rate after R1vasc than after R0 resection in patients with CRLM, without a corresponding overall-survival difference [14], and molecular data suggest that the effect of margin phenotype may vary with tumor biology [15]. R1vasc should therefore be regarded as a selected parenchymal-preserving strategy rather than a substitute for R0 resection.

The vessel-skeletonized parenchymal-sparing hepatectomy (VESPAH) concept was developed for extensive bilobar CRLM to achieve complete macroscopic clearance while preserving intrahepatic inflow, outflow, and functional liver volume [16]. Its initial series was small and retrospective, but it illustrates a broader question that extends beyond the R0/R1 debate: how does the index operation influence the patient’s capacity to receive subsequent systemic therapy and repeat local treatment? This question has become more relevant as repeat hepatectomy [7] and thermal ablation for selected small CRLM [17] have expanded the options for repeated liver-directed treatment.

Recent reviews have addressed margin width and the oncological rationale for R1 vascular hepatectomy in detail [18]. The present review therefore focuses on a different issue: the relationship between margin-aware parenchymal preservation and the subsequent treatment trajectory. We discuss the evolution from margin width to margin phenotype, the anatomical basis and limits of tumor–vessel detachment, and the role of PSH in preserving liver function and future local-treatment options. We use the term “treatment continuity” to describe preservation of the physiological and anatomical capacity to receive indicated systemic therapy and repeat curative-intent local treatment over the disease course.

The literature discussed in this focused narrative review was identified through PubMed searches conducted up to 20 September 2026, using combinations of the terms “colorectal liver metastases”, “surgical margin”, “R1 vascular”, “vascular detachment”, “parenchymal-sparing hepatectomy”, “repeat hepatectomy”, and “thermal ablation”. Reference lists of key original studies and relevant reviews were also examined. Priority was given to studies directly addressing margin phenotype, parenchymal preservation, local recurrence, subsequent liver-directed treatment, and clinically relevant long-term outcomes. The review was intended as a focused conceptual synthesis rather than an exhaustive systematic review.

## 2. From Margin Width to Margin Phenotype and Biology

### 2.1. From the One-Centimeter Rule to a Microscopically Negative Margin

The traditional one-centimeter rule arose from early series in which a margin of less than 1 cm was associated with inferior survival [19]. Histological work subsequently showed that microscopic extension beyond the visible edge of CRLM is usually confined to the immediate peritumoral liver, supporting a narrower threshold [20]. Large clinical series then separated margin width from margin positivity: among patients with R0 resection, survival differences across margin-width categories were inconsistent, whereas a microscopically positive margin remained associated with local recurrence and worse outcome [1]. A propensity-matched analysis further suggested that a cancer-free margin of 1 mm could provide outcomes comparable to wider margins [2].

The 1 mm threshold is nevertheless a minimum rather than a universal optimum. A meta-analysis of R0 resections found a stepwise association between wider margins and improved recurrence-free and overall survival [3], and contemporary population-based data continue to support a prognostic advantage for margins of at least 1 mm [4]. These observations are not contradictory. They support two practical principles: tumor exposure at the transection surface should be avoided, and a wider margin is desirable when it can be obtained without meaningful additional anatomical cost. The clinically relevant question is therefore not whether wider is better in the abstract, but what must be sacrificed to obtain that width in a particular patient.

A further methodological problem is that “margin” is not measured uniformly. Some studies define R1 as tumor at the inked surface, whereas others use a distance of less than 1 mm; patient-level analyses may classify an entire operation as R1 on the basis of a single involved area, while lesion-level analyses treat each metastasis separately. In patients undergoing multiple parenchymal-sparing resections, these conventions can produce markedly different estimates of risk. Any contemporary interpretation of margin studies must therefore consider not only the measured width but also the definition, unit of analysis, and treatment era from which that width was derived.

### 2.2. Margin Status in the Era of Systemic Therapy and Molecular Stratification

Modern chemotherapy has not eliminated the prognostic relevance of margin status. Cohort studies using contemporary regimens continue to identify R1 resection as an adverse factor [21], and population-based analyses suggest that a margin of at least 1 mm retains prognostic value even in the modern chemotherapy era [4]. At the same time, the apparent effect of margin is strongly confounded by tumor number, size, bilobar distribution, extrahepatic disease, and response to systemic therapy—all of which influence both prognosis and the technical likelihood of achieving a wide margin [22,23]. Patients with narrow or positive margins are therefore not a random sample of all resected patients; they frequently have more complex and biologically unfavorable disease. Margin status reports on both the operation and the disease selected for that operation, and observational studies cannot completely separate these components.

Molecular analyses further support a biology-dependent interpretation. In one series, KRAS-mutant tumors with a margin of less than 5 mm had substantially higher local recurrence than those with wider margins, whereas the same pattern was not evident in KRAS wild-type disease [24]. More recent multicenter data examining R1 phenotype according to KRAS and BRAF status likewise suggest that the prognostic effect of margin type is not uniform [15]. These findings do not establish a genotype-specific margin threshold, nor do they justify accepting a positive margin in biologically aggressive disease. Rather, they caution against assuming that the same number of millimeters has the same clinical meaning in every patient. R0 resection remains the appropriate goal; the practical issue is how much additional liver and vascular architecture should be sacrificed when the incremental oncological value of a wider margin is uncertain and the competing risk of systemic recurrence is high.

## 3. Anatomical Re-Definition of the R1 Margin: R1par Versus R1vasc

### 3.1. Two Anatomically Distinct R1 Events

A microscopically positive margin can arise in two different settings. In R1par, tumor is exposed on the raw parenchymal transection surface because the plane passes through or immediately adjacent to the lesion. In R1vasc, the tumor is deliberately detached from the surface of a major intrahepatic vessel that is preserved in the remnant, leaving no parenchymal cuff at that interface. Conventional pathology may label both situations as R1, but their operative intent and anatomical substrate differ.

Terminology in this field is not yet standardized, and the present review adopts a single convention throughout. R1par denotes microscopic tumor exposure on a divided parenchymal surface, whatever the structure nearby; R1vasc denotes planned detachment of tumor from the wall of a major intrahepatic vessel that is preserved in the remnant, whether that vessel is a hepatic vein or a Glissonean pedicle. Where a cited study drew the boundary differently, this is stated at the point of citation. The most important such difference concerns the original VESPAH report, in which detachment from a main Glissonean pedicle was classified as R1-Par and R1-Vas was reserved for detachment from a hepatic vein trunk [16]; figures from that series are therefore not interchangeable with figures from studies using the convention adopted here.

The clinical behavior also appears to differ. In the index study separating these entities, local recurrence risk did not differ significantly between R1vasc and R0 resection (3.9% vs. 1.5% per resection area), whereas it was significantly higher after R1par resection (13.6%) [11]. Subsequent work from the same group suggested that selected tumor–vessel detachment and hepatic-vein preservation at the caval confluence could be achieved without the same oncological penalty observed after parenchymal exposure [12]. A survey of hepatopancreatobiliary surgeons showed that this distinction is already used in clinical decision-making despite the absence of a standardized reporting system [13]. The studies that have shaped this reinterpretation of the margin are summarized in Table S1.

This distinction also exposes a limitation of patient-level R0/R1 reporting. A patient with ten resection areas may have nine clearly negative parenchymal margins and one planned detachment from a hepatic vein; classifying the entire operation as “R1” loses the anatomical information most relevant to local recurrence. Conversely, grouping an unplanned parenchymal exposure with a deliberate vascular detachment can dilute the adverse signal of R1par and make R1vasc appear less favorable than it is in carefully selected cases. For studies of multiple CRLM, resection-area-level reporting is therefore particularly important.

### 3.2. Anatomical Basis and Limits of Tumor–Vessel Detachment

The rationale for R1vasc depends on a reproducible dissection plane between tumor and vessel. Laennec’s capsule forms a fibrous interface around the hepatic parenchyma and vascular structures; surgical approaches based on this layer have demonstrated that it can be followed around Glissonean pedicles and hepatic veins [25,26]. When a metastasis compresses rather than invades a vessel, this interface may permit macroscopic separation while preserving the vessel. The resulting surface is fundamentally different from unplanned exposure within divided liver parenchyma.

The practical importance of the layer is greatest around major hepatic veins, where sacrificing the vessel may convert a limited resection into a much larger anatomical operation. Around Glissonean structures, however, the decision is more constrained because pedicle involvement can extend along portal, arterial, and biliary components. The presence of upstream bile-duct dilatation is therefore a particularly important warning sign that simple detachment may be oncologically inadequate [16]. The same “R1vasc” terminology can thus conceal different anatomical situations, and the involved structure should be specified whenever possible.

This distinction is conditional, not absolute. Detachment should be considered only when preoperative imaging and intraoperative ultrasonography support contact or compression without convincing vascular invasion. Deformity, stenosis, circumferential encasement, or broad hepatic-vein involvement argues against detachment; segmental bile-duct dilatation adjacent to a tumor contacting a Glissonean pedicle similarly suggests invasion and favors anatomical resection of the involved territory [16]. Thus, R1vasc is best understood as an anatomically selected endpoint, not a general license to accept a narrow margin.

The vascular structure involved also matters. Detachment from a hepatic vein and dissection along a Glissonean pedicle are not necessarily equivalent, because venous walls, pedicle sheaths, adjacent bile ducts, and patterns of microscopic extension differ. Terminology is not uniform across studies. In the original VESPAH report, R1-Par included both tumor exposure at the parenchymal transection surface and detachment from a main Glissonean pedicle, whereas R1-Vas was reserved for detachment from a hepatic vein trunk [16]. This terminological heterogeneity is one reason why pooled R1 analyses are difficult to interpret and why the involved structure, operative intent, and margin definition should be reported explicitly.

### 3.3. Current Oncological Evidence and Interaction with Tumor Biology

The evidence base remains observational and heterogeneous. A recent meta-analysis restricted to minimally invasive liver surgery found a higher local recurrence rate after R1vasc than after R0 resection in patients with CRLM, without a corresponding overall-survival difference [14]. This may indicate a real but potentially salvageable local risk, or it may reflect variation in case selection and the accuracy with which compression is distinguished from invasion. Either interpretation emphasizes that technical reproducibility and careful selection are central to the oncological adequacy of detachment.

Biological stratification adds further nuance. In a cohort of patients with known KRAS status, R1par was associated with more local recurrence than R1vasc, while the relative behavior of R1vasc and R0 varied by genotype [27]. More recently, a multicenter cohort of 1078 patients with documented KRAS and BRAF status found worse overall survival after R1par than after R0, with the largest detriment in KRAS-mutant tumors, whereas R1vasc was not associated with a significant survival decrement across molecular subgroups [15]. These data remain retrospective and do not justify routine R1vasc, but they support a graded interpretation of margin phenotype: R1par should be avoided whenever technically possible, while R1vasc may be considered in carefully selected patients when it permits complete macroscopic clearance while preserving major vascular architecture.

Three features of these cohorts make a causal reading of the favorable R1vasc results unsafe. First, detachability is itself a selection variable: a tumor that can be peeled from a venous wall along an intact plane is, by that fact, one that has not invaded it, and in many cases it is also disease that responded to preoperative chemotherapy, a point returned to in Section 4.2. The margin phenotype therefore reports in part the anatomy and biology that permitted it. Second, the decision to detach rather than resect is taken by the operating surgeon at the time of operation, so operative intent, technical confidence and case mix are confounded with the exposure under study; this is confounding by indication, and no amount of multivariable adjustment within a retrospective series removes it. Third, the comparison groups are not exchangeable, because patients in whom detachment was not attempted include both those with genuine vascular invasion and those treated before the technique was adopted at that center.

The molecular data illustrate the same problem from the opposite direction: the absence of a survival decrement after R1vasc across KRAS and BRAF subgroups [15] is reassuring, but it cannot distinguish a genuinely benign margin phenotype from the preferential allocation of favorable anatomy to detachment. Establishing that R1vasc is oncologically safe therefore requires designs in which the anatomical scenario, rather than the margin obtained, defines the comparison groups.

## 4. Margin-Aware Parenchymal-Sparing Surgery for Multiple Bilobar CRLM

### 4.1. Where Margin and Parenchymal Preservation Genuinely Compete

For most peripheral CRLM, an adequate parenchymal margin can be obtained at little functional cost. The conflict becomes clinically important in two settings: a deep lesion contacting a major Glissonean pedicle or hepatic vein, and extensive bilobar disease in which the sum of conventional anatomical resections would leave an inadequate remnant. In such situations, the alternatives are not simply “wide” versus “narrow” margins, but different strategies for achieving complete macroscopic clearance while preserving enough functional liver and vascular architecture for the rest of the treatment course.

**Figure 1:**
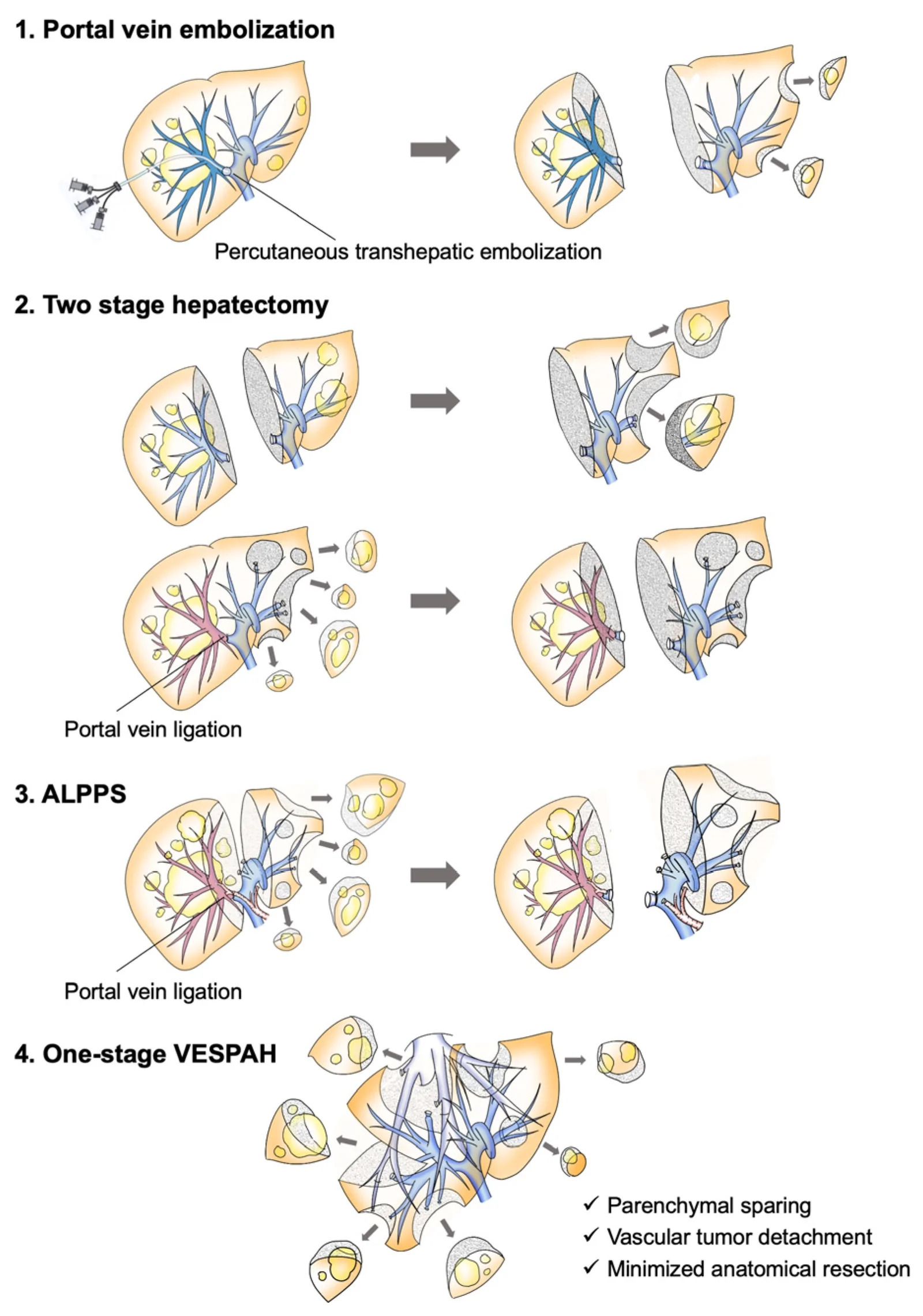
Strategies for multiple bilobar colorectal liver metastases. (1) Portal vein embolization followed by hepatectomy after hypertrophy of the future liver remnant. (2) Two-stage hepatectomy, shown in its classical form (upper row) and with portal vein ligation combined with clearance of the future liver remnant at the first stage (lower row). (3) Associating liver partition and portal vein ligation for staged hepatectomy (ALPPS). (4) One-stage vessel-skeletonized parenchymal-sparing hepatectomy (VESPAH): minimal anatomical and non-anatomical resections that preserve parenchyma and the intrahepatic vascular skeleton, with detachment and preservation of involved hepatic veins where an anatomical plane exists and resection of the corresponding territory where a Glissonean pedicle is invaded. Strategies (1–3) require an interstage interval during which disease progression or treatment-related dropout may occur, whereas strategy (4) aims to achieve clearance in a single stage. Throughout the figure, liver parenchyma is shown in orange, metastases in yellow, hepatic veins in blue, the portal vein in pink, and tissue to be resected in grey stipple. Reproduced with permission from Ref. [32]. 2023, Herusu Publishing Co., Inc.

The principle that narrow or microscopically positive margins may sometimes be accepted by necessity in extensive bilobar disease has long been recognized [28]. Staged strategies remain essential when one-stage clearance is not safe, but they have limitations: a proportion of patients undergoing two-stage hepatectomy never reach the second stage, usually because of progression or inadequate hypertrophy [29], while ALPPS trades faster hypertrophy for greater procedural complexity and non-negligible morbidity [30]. Single-stage ultrasonography-guided parenchymal-sparing strategies have provided an alternative in selected patients [31]. These options are compared schematically in Figure 1.

**Figure 2:**
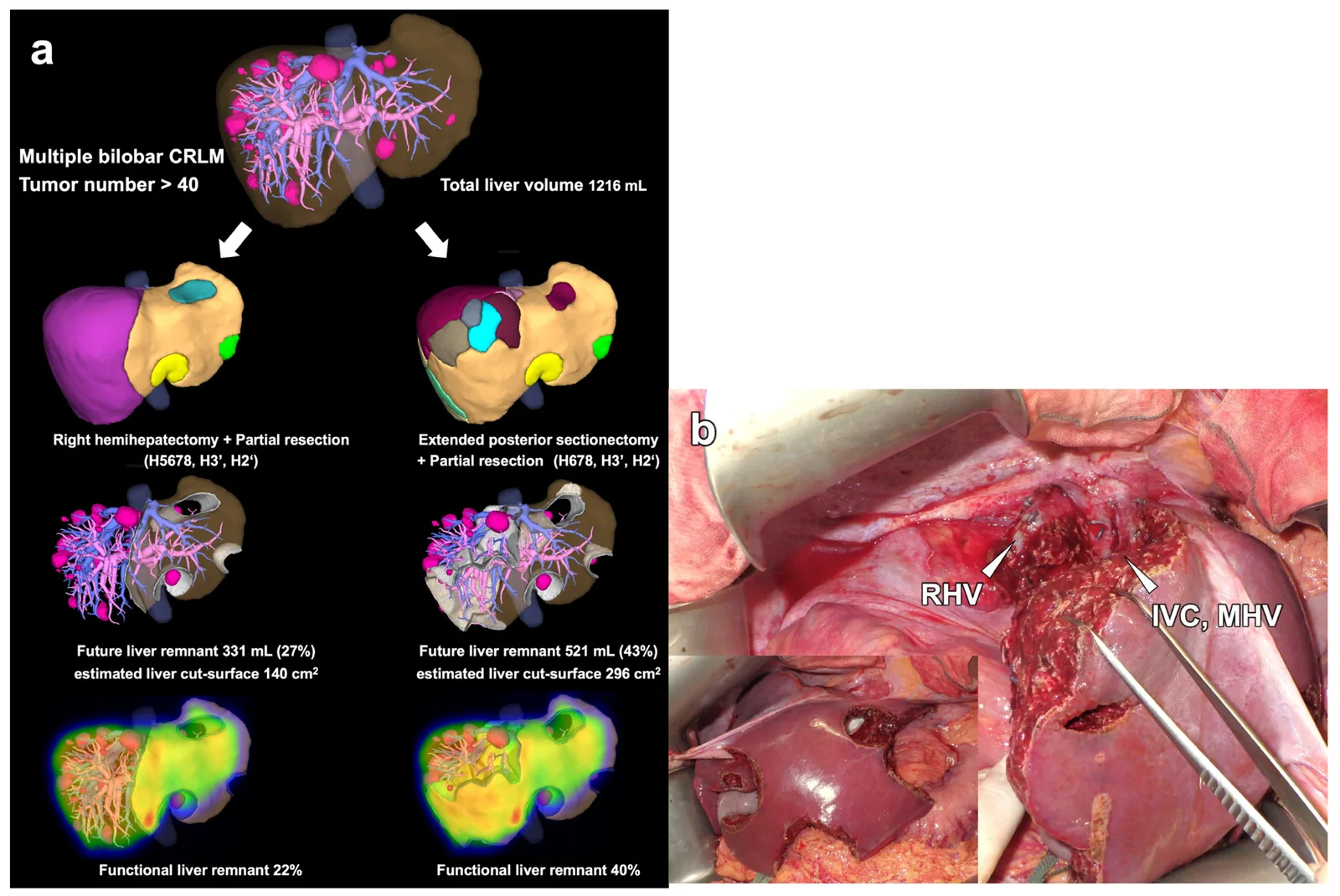
Preoperative simulation used to avoid major hepatectomy in a patient with more than 40 bilobar colorectal liver metastases (total liver volume 1216 mL). (**a**) Three-dimensional simulation comparing right hemihepatectomy combined with partial resections and extended posterior sectionectomy combined with partial resections. The parenchymal-sparing plan increased the future liver remnant from 331 mL (27%) to 521 mL (43%) and the functional liver remnant from 22% to 40%, while the estimated cut surface increased from 140 to 296 cm^2^. (**b**) Intraoperative appearance after the parenchymal-sparing procedure: the right hepatic vein and the confluence of the inferior vena cava and middle hepatic vein are skeletonized on the cut surface, the tumor having been detached from the venous adventitia (R1 vascular). Inset: the multiple limited transection surfaces distributed over the remnant. IVC, inferior vena cava; MHV, middle hepatic vein; RHV, right hepatic vein. Reproduced with permission from Ref. [32]. 2023, Herusu Publishing Co., Inc.

This is also the setting in which the consequences of the first operation are magnified. A conventional major hepatectomy can simplify the immediate clearance of one hemiliver but may leave little capacity for subsequent surgery in the remnant, whereas a one-stage PSH strategy distributes the technical burden across multiple limited transection planes. Neither approach is universally superior. The choice depends on whether all macroscopic disease can be cleared safely, whether major vessels must truly be sacrificed, and whether the expected remnant retains sufficient reserve for both recovery and possible future treatment. The magnitude of this trade-off can be quantified before operation. In a representative patient with more than 40 bilobar metastases, simulation compared right hemihepatectomy with extended posterior sectionectomy combined with multiple partial resections: the parenchymal-sparing plan raised the projected remnant from 27% to 43% of total liver volume and the functional remnant from 22% to 40%, at the cost of more than doubling the total transection surface area, from 140 to 296 cm^2^ (Figure 2).

### 4.2. Vessel Skeletonization as Navigation and Margin Definition

VESPAH was developed within this clinical space [16]. Its empirical basis is a single-center retrospective series of 20 patients, so the account that follows describes a technique and the decision rules behind it rather than evidence of comparative efficacy. It is best regarded not as a separate named operation but as a set of decision rules for conducting PSH around the preserved intrahepatic vascular skeleton. Skeletonization serves two purposes. First, the ultrasonographically defined vascular tree provides a coordinate system for locating multiple deep lesions and for navigating an irregular transection plane [33]. Second, where a lesion contacts a preserved vessel, the vessel–tumor interface itself defines the margin; elsewhere, a conventional parenchymal margin is obtained. A single operation can therefore contain several distinct margin phenotypes, supporting lesion- or resection-area-level rather than patient-level reporting.

In practice, the strategy combines non-anatomical partial resections with the smallest anatomical resection required by true pedicle invasion. Deep lesions are approached along skeletonized vessels rather than by repeated blind wedge resections, and the transection surface may be deliberately curved or polymorphic to connect multiple target lesions while preserving major inflow and outflow. When a higher-order Glissonean branch is invaded, segmental or subsegmental anatomical resection can be incorporated; more proximal pedicle involvement requires a correspondingly larger anatomical unit [16,34]. The aim is not to maximize the number of limited resections, but to match the extent of resection to the minimum anatomy required for complete clearance.

The original VESPAH description grouped these procedures according to the smallest anatomical unit required for clearance: multiple non-anatomical partial resections when vessels could be preserved, limited segmental resection when more distal pedicles were involved, and larger sectional resection when proximal pedicles required division [16]. These categories are less important as named procedures than as evidence of a governing principle: the vascular skeleton is preserved until tumor anatomy provides a specific reason to sacrifice it. This principle allows the transection plane to be adapted lesion by lesion rather than forcing all disease into a predefined flat anatomical plane.

**Figure 3:**
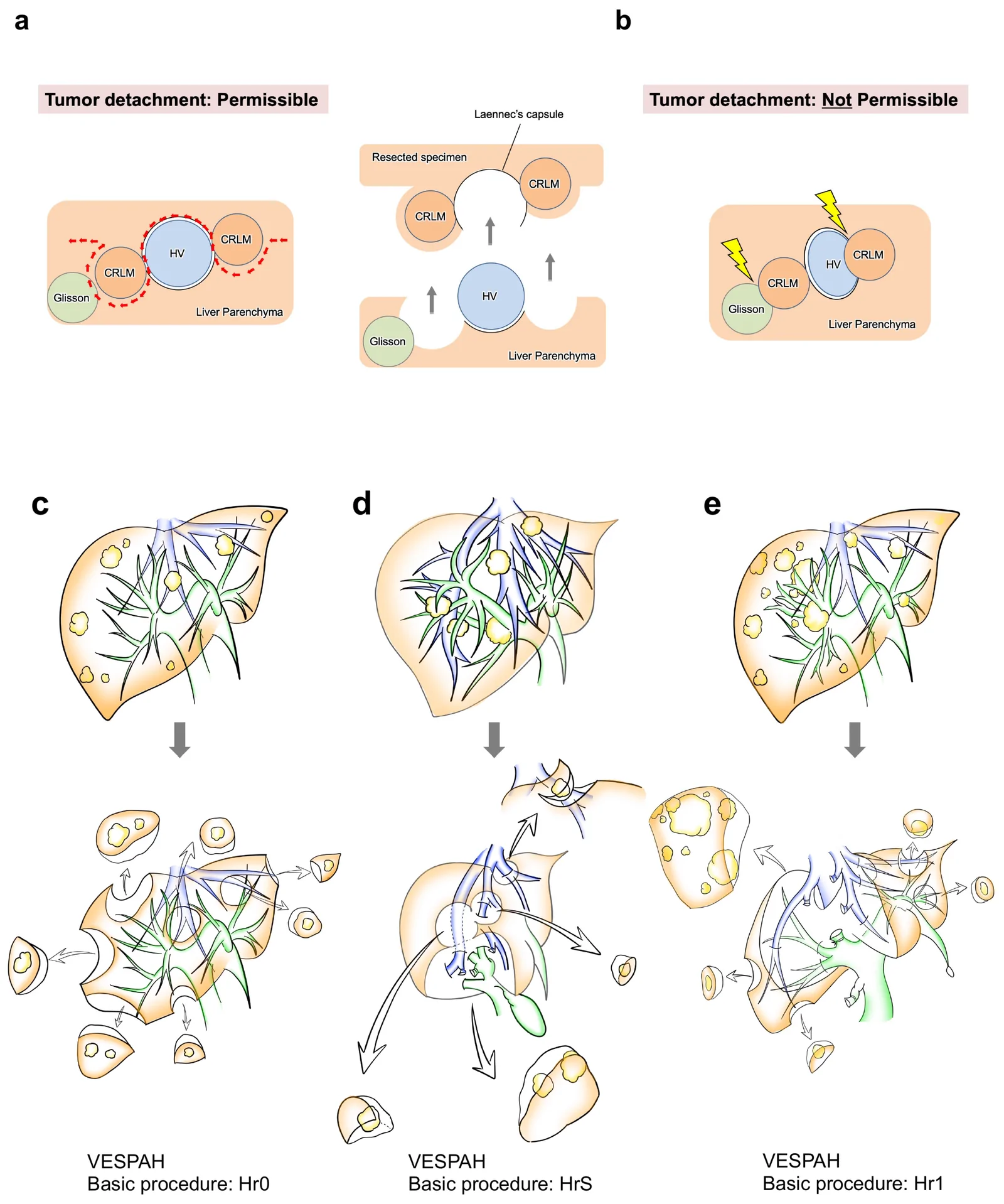
Permissibility of tumor–vessel detachment and the resulting basic procedures in vessel-skeletonized parenchymal-sparing hepatectomy (VESPAH). (**a**) Detachment is permissible: the metastasis compresses the hepatic vein and/or Glissonean pedicle without invading it, and the lesion can be separated along Laennec’s capsule, leaving the intrahepatic structures skeletonized on a polygonal cut surface. The dotted line indicates the transection line. (**b**) Detachment is not permissible: tumors invading a Glissonean pedicle with bile-duct dilatation, or involving a hepatic vein with broad adhesion or stenotic deformity, require resection of the corresponding structure. (**c**–**e**) The three basic procedures that follow from these rules, classified by the smallest anatomical unit required for clearance: (**c**) multiple non-anatomical partial resections (Hr0); (**d**) limited anatomical segmentectomy (HrS); and (**e**) limited anatomical sectionectomy (Hr1). In panels (**c**–**e**), hepatic veins are shown in blue, Glissonean pedicles in green, metastases in yellow, and the resected specimens in grey stipple. Each may be combined with additional partial resections. Panels (**a**,**b**) are reproduced from Umeda Y et al. Langenbecks Arch. Surg. 2022, 407, 685–697 [16], under the Creative Commons Attribution 4.0 International License (http://creativecommons.org/licenses/by/4.0/, accessed on 26 October 2026); panels (**c**–**e**) are reproduced with permission from Ref. [32]. 2023, Herusu Publishing Co., Inc.

The central decision is whether a contacted vessel can be preserved (Figure 3a,b). A tumor that compresses a vessel without deformation or stenosis may be detached if an intact plane can be followed. Glissonean invasion, especially when associated with peripheral bile-duct dilatation, requires resection of the minimum anatomical territory supplied by the involved pedicle [16,34]. Broad hepatic-vein adhesion, deformity, or stenosis favors venous resection; preservation without reconstruction may still be possible when collateral drainage through communicating veins or an inferior right hepatic vein is demonstrated [35]. Importantly, apparent vascular attachment can regress after preoperative chemotherapy, so detachability should be reassessed on post-treatment imaging and confirmed intraoperatively [36].

**Figure 4:**
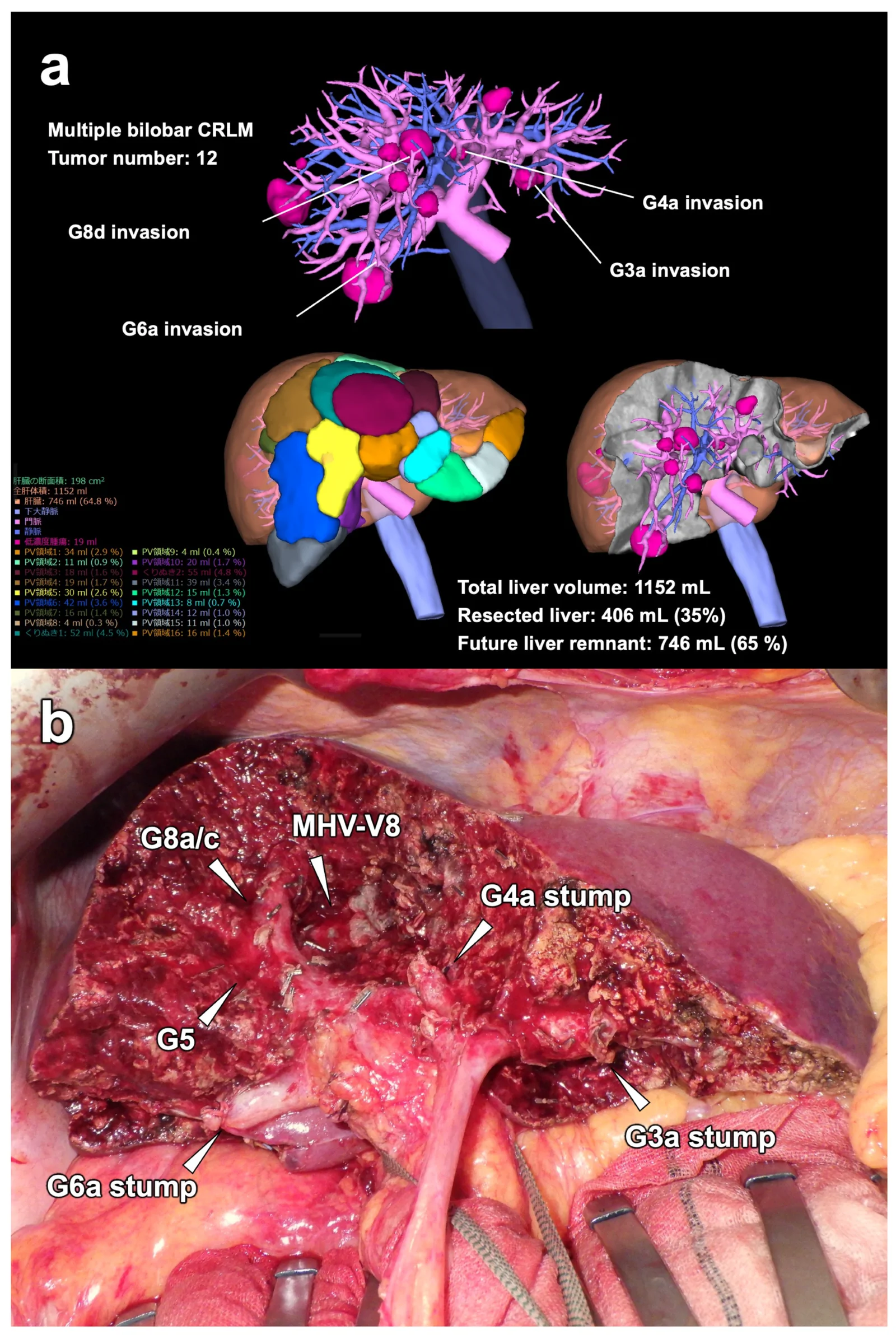
Preoperative simulation reproduced at operation in a patient with 12 bilobar colorectal liver metastases (total liver volume 1152 mL; resected volume 406 mL, 35%; future liver remnant 746 mL, 65%; estimated cut surface 198 cm^2^). (**a**) Simulation identifying Glissonean invasion at S3a, S4a, S6a and S8d, with the corresponding portal territories color-coded. (**b**) Transection surface showing the divided G3a, G4a and G6a pedicles together with the preserved G8a/c and G5 pedicles and the V8 tributary of the middle hepatic vein. Only the invaded pedicles were sacrificed; the remaining vascular skeleton was preserved and is directly identifiable on the cut surface. MHV, middle hepatic vein. The color key in panel (**a**) is an unmodified screen output of the three-dimensional simulation software and is labelled in Japanese; its terms are, in order, liver cut-surface area, total liver volume, liver (future remnant) volume, inferior vena cava, portal vein, hepatic vein, hypodense tumor, portal vein territory 1–16, and carved-out (partial resection) volume 1 and 2. Reproduced with permission from Ref. [32]. 2023, Herusu Publishing Co., Inc.

**Figure 5:**
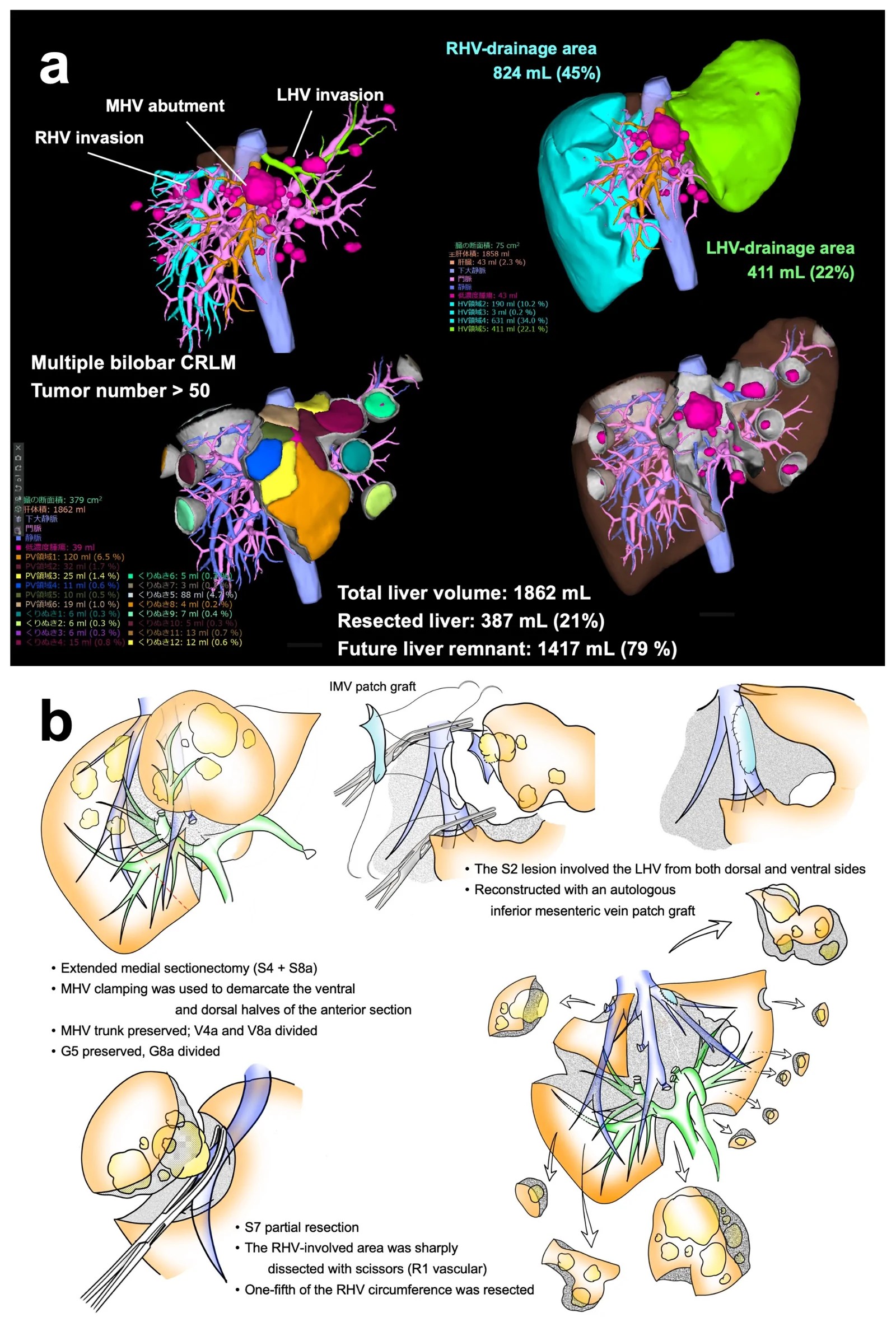
The case shown in Video S1: more than 50 bilobar colorectal liver metastases (total liver volume 1862 mL; resected volume 387 mL, 21%; future liver remnant 1417 mL, 79%; estimated cut surface 379 cm^2^). (**a**) Preoperative simulation showing invasion of the right and left hepatic veins and abutment of the middle hepatic vein, with the right and left hepatic venous drainage territories (824 mL, 45% and 411 mL, 22%) delineated. (**b**) Operative plan: extended medial sectionectomy (S4 + S8a) with preservation of the middle hepatic vein trunk; sharp detachment at the right hepatic vein (R1 vascular) with resection of one-fifth of its circumference and suture closure; and resection of two-thirds of the left hepatic vein circumference with autologous inferior mesenteric vein patch reconstruction, together with the multiple limited resections that completed the clearance. IMV, inferior mesenteric vein; LHV, left hepatic vein; MHV, middle hepatic vein; RHV, right hepatic vein. The two color keys in panel (**a**) are unmodified screen outputs of the three-dimensional simulation software and are labelled in Japanese; their terms are liver cut-surface area, total liver volume, liver volume, inferior vena cava, portal vein, hepatic vein, hypodense tumor, hepatic vein drainage territory 2–5, portal vein territory 1–6, and carved-out (partial resection) volume 1–12; the right hepatic venous drainage territory quoted above is the sum of hepatic vein territories 2–4. Reproduced with permission from Ref. [32]; 2023, Herusu Publishing Co., Inc.

The procedural patterns that follow from these rules—multiple non-anatomical partial resections, limited anatomical segmentectomy, and limited anatomical sectionectomy, each combinable with additional partial resections—are determined lesion by lesion rather than by a predefined resection template (Figure 3c–e). Because the plan is anatomical rather than improvised, it can be simulated before operation and reproduced at the transection surface, where the divided and preserved pedicles become directly identifiable (Figure 4). Figure 5 and Video S1 show a patient with more than 50 metastases in whom complete clearance was achieved with removal of 21% of the liver volume, including detachment from the right hepatic vein and circumferential resection with patch reconstruction of the left hepatic vein; they are included as technical illustration and are not discussed further here. VESPAH names one specific implementation, but the decision rules it formalizes—vessel-defined margins, lesion-level margin reporting, and resection to the minimum anatomical unit required for clearance—are applicable wherever parenchymal preservation is attempted.

### 4.3. Technical Trade-Offs and Boundaries

Parenchymal preservation is not without technical cost. In the VESPAH series, repeated dissection around major structures and the irregular transection surface were associated with longer cumulative inflow occlusion than major hepatectomy [16]. Skeletonization also exposes a larger length of Glissonean sheaths and venous walls, increasing the technical demands of hemostasis and bile-duct protection. These features may increase operative complexity even when total blood loss or major morbidity is not clearly higher. They reinforce the requirement for high-quality intraoperative ultrasonography, familiarity with segmental vascular anatomy, and a low threshold for abandoning detachment when the anatomical plane is uncertain. Further procedural detail is available in the original report [16].

Equally important are the limits of the strategy. Extensive invasion of a major Glissonean pedicle, non-detachable hepatic-vein involvement, or an inadequate projected remnant remains an indication for anatomical or major hepatectomy, vascular resection when appropriate, or a staged strategy. No comparative study of adequate size has tested whether these decision rules improve outcome; they are offered as a reproducible way of making the trade-off explicit, not as a validated standard. Margin-aware PSH is therefore not an argument against major hepatectomy; it is an argument that every vessel and segment of functioning parenchyma sacrificed for additional margin should have a demonstrable oncological or technical justification.

## 5. Beyond the Margin: Treatment Continuity and Future Salvageability

### 5.1. The Treatment Course, Not the Index Operation, Is the Relevant Time Horizon

Recurrence remains common after macroscopically complete resection of CRLM, and the liver remnant is one of the most frequent sites. For many long-term survivors, the therapeutic history therefore consists of several episodes of treatment rather than one curative event. Systemic therapy, repeat hepatectomy, ablation, and treatment of extrahepatic recurrence may all contribute at different time points. The condition of the patient and of the liver after the initial hepatectomy therefore matters beyond perioperative safety. The relevant question is not only whether the present disease has been cleared, but whether the operation preserves the capacity to deliver indicated therapy when recurrence or residual systemic risk emerges.

Randomized trials of perioperative or postoperative chemotherapy illustrate why this broader perspective is needed. EORTC 40983 improved progression-free survival with perioperative FOLFOX without demonstrating an overall-survival benefit [37]. JCOG0603 similarly showed an improvement in disease-free survival with postoperative mFOLFOX6, but long-term follow-up did not demonstrate an overall-survival advantage [38,39]. These trials should not be interpreted as evidence that postoperative systemic therapy is unimportant; rather, the discordance between recurrence-related endpoints and overall survival indicates that long-term outcome after CRLM resection is influenced by treatment delivered after recurrence as well as by the index operation and the first postoperative regimen. We use “treatment continuity” to describe preservation of the physiological and anatomical capacity to receive indicated systemic and local therapies over the subsequent disease course. It is a property of the treatment trajectory, not a claim that any specific therapy must be delivered to every patient.

### 5.2. How the Extent of Hepatectomy Can Constrain Subsequent Treatment

Three mechanisms link the index operation to the subsequent treatment trajectory. First, preservation of functional reserve reduces the risk of clinically relevant posthepatectomy liver failure (PHLF), a standardized postoperative complication defined and graded by the ISGLS according to postoperative hepatic dysfunction and its impact on clinical management [40]. This is particularly relevant after oxaliplatin- or irinotecan-based chemotherapy, which can produce sinusoidal injury or steatohepatitis in otherwise non-cirrhotic liver [41,42]. The relationship is not simply volumetric: the same remnant volume may behave differently according to chemotherapy exposure, steatosis, age, and baseline function. Future-liver-remnant volume therefore remains only one part of the functional calculation. Standardized volumetry is indispensable when major resection is contemplated [43], but it does not fully describe a liver exposed to systemic therapy, and in selected high-risk cases additional functional assessment may help to characterize reserve beyond size alone [44]. In this sense, a parenchymal-sparing strategy preserves both parenchymal volume and, potentially, functional reserve, and reduces dependence on predicting how close the remnant can safely be pushed to its functional limit.

Second, postoperative morbidity can disrupt the intended sequence of multimodal therapy. In an analysis of patients undergoing CRLM resection, initiation of postoperative chemotherapy beyond eight weeks was more frequent among patients who experienced postoperative complications [45]. Such observational associations are confounded by disease burden and patient fitness and should not be interpreted as evidence that a specific timing threshold improves overall survival. The direction of causation cannot be established from such data, because the complication and the delay may both follow from more extensive disease and a less fit patient. The association is reported here for a narrower purpose: to indicate that delivery of planned treatment is not independent of the perioperative course.

Third, the volume and vascular architecture of the remnant determine whether another liver-directed intervention will be technically possible at recurrence. This is the most specific link between parenchymal preservation and treatment continuity. A remnant reduced to the minimum required for recovery from the first operation may be anatomically unsuitable for a second hepatectomy even if recurrent disease is otherwise favorable. The lost option becomes visible only when it is needed.

### 5.3. The VESPAH Cohort as a Proof-of-Concept

The VESPAH series provides an illustrative, hypothesis-generating example. Among 33 patients with eight or more bilobar metastases, 20 underwent VESPAH and 13 underwent major hepatectomy [16]. The groups were small and unmatched, and the distribution of disease across liver segments differed, but the burden was sufficiently advanced in both groups to make preservation of future treatment options clinically meaningful. Clinically relevant PHLF occurred in 5% of the VESPAH group versus 38% of the major-hepatectomy group, and postoperative chemotherapy was initiated within two months in 90% versus 38%. Among patients with intrahepatic recurrence, repeat hepatectomy was incorporated into treatment in 86% after VESPAH and 57% after major hepatectomy; seven patients in the VESPAH group underwent three hepatectomies during their disease course, compared with none in the major-hepatectomy group.

Margin phenotype differed between the groups, as the strategies imply. Using the categories of the source study, the final margin status in the VESPAH group was R0 in 9 patients (45%), R1-Par in 3 (15%) and R1-Vas in 8 (40%), against R0 in 12 (92%), R1-Par in 1 (8%) and R1-Vas in none of the major-hepatectomy group (*p* = 0.015) [16]. Hepatic local recurrence was 8.3% for R0, 33% for R1-Par and 0% for R1-Vas (*p* = 0.269), and no patient developed recurrence at a site where tumor had been detached from a major hepatic vein. Two caveats are essential, and both illustrate the reporting problem set out in Section 3.1. The classification was made per patient rather than per resection area, so the number of individual detachment sites at risk cannot be recovered from the published data; and R1-Par in that series included detachment from a main Glissonean pedicle, a category treated separately in the present review. With 3 and 8 patients in the respective R1 strata, the absence of recurrence at detachment sites is compatible with a wide range of true event rates and does not by itself establish the oncological safety of detachment.

These data should not be interpreted as evidence of a survival advantage. Median recurrence-free survival was eight months in both groups, and overall survival did not differ significantly. The comparison was retrospective, small, unmatched, and subject to indication bias. Its value is narrower: it illustrates how two operations applied to extensive disease can differ markedly in postoperative liver failure, return to systemic therapy, and eligibility for further liver-directed treatment even when disease control at the index operation is similar. Those downstream differences are not captured by margin status alone.

### 5.4. Repeat Hepatectomy and Thermal Ablation as Future Options

The strongest direct evidence for the value of parenchymal preservation comes from repeat hepatectomy. In a single-center retrospective cohort of 300 patients with a solitary metastasis of 30 mm or less, repeat hepatectomy was performed in 68% after PSH compared with 24% after non-parenchymal-sparing resection, and non-PSH was an independent risk factor for non-candidacy for repeat hepatectomy on multivariate analysis (hazard ratio 8.18) [7]. Importantly, in that cohort the gain in salvageability was not accompanied by an increase in recurrence within the remnant liver. Pooled data are less uniform: a meta-analysis of 22 studies including 7228 patients found higher positive-margin and intrahepatic-recurrence rates after parenchymal-sparing resection, although liver recurrence-free survival and overall and disease-free survival did not differ significantly between approaches [9]. This trade-off should be acknowledged. Parenchymal preservation may incur a local-control cost in some settings; its potential oncological value therefore lies not in preventing intrahepatic recurrence, but in preserving the feasibility of repeat local treatment when recurrence occurs. Third and subsequent hepatectomies can provide acceptable outcomes in carefully selected patients [46], and surgical treatment of recurrence after complex staged strategies has likewise been associated with prolonged survival, although all such comparisons are subject to substantial selection bias [47]. The central point is anatomical: preserving parenchyma and vascular inflow/outflow at the index operation may preserve the feasibility of local treatment for future recurrences that might otherwise become technically untreatable.

Thermal ablation extends the concept of parenchymal preservation beyond resection. In the COLLISION randomized trial, which enrolled 296 patients with up to 10 CRLM each smaller than 3 cm, thermal ablation met the prespecified criterion for non-inferiority to resection in overall survival (hazard ratio 1.05, 95% confidence interval 0.69–1.58), with a more favorable safety profile [17]. These data qualify rather than overturn the traditional hierarchy in which ablation is reserved for lesions that cannot be resected, and three limits on their reach should be stated. The finding applies only to small, resectable and ablatable lesions; at a median follow-up of 28.9 months the long-term oncological outcomes remain immature; and the trial does not address the larger or vessel-adjacent lesions that generate the decisions discussed in this review. In contemporary multimodal planning, ablation can complement resection for selected small lesions when doing so avoids unnecessary parenchymal loss. Its oncological adequacy is itself margin-dependent, however, because insufficient minimal ablative margins are associated with local tumor progression [48]. Resection margin and ablative margin are therefore different technical measurements serving the same principle: complete local treatment with the least necessary anatomical expenditure. Proximity to major vessels, bile ducts, or other vulnerable structures remains relevant because it may prevent an adequate ablative margin from being achieved safely.

### 5.5. Making Treatment Continuity Measurable

If treatment continuity is clinically relevant, it should be reported rather than inferred. Existing composite quality measures provide a precedent. Textbook outcome after CRLM surgery has been associated with long-term survival [49]. In the liver-first setting for synchronous CRLM, a procedure-specific definition has further incorporated full completion of the planned multimodal treatment sequence [50]. More broadly, textbook outcome in liver surgery has been developed as a multidimensional composite measure of perioperative quality [51]. Yet current surgical comparisons still focus mainly on margin status, complications, recurrence-free survival, and overall survival. These endpoints do not capture whether a patient who develops recurrence remains technically treatable. For studies comparing PSH with major hepatectomy, we propose reporting four readily obtainable measures in addition to conventional endpoints: (i) clinically relevant PHLF; (ii) time to initiation or resumption of planned systemic therapy; (iii) the proportion of patients with hepatic recurrence who undergo subsequent curative-intent local treatment; and (iv) the cumulative number of liver-directed interventions during follow-up. Table 1 summarizes what the principal comparative studies of parenchymal preservation report about the subsequent treatment course, and shows how unevenly these outcomes are currently captured.

These measures are descriptive, not validated surrogate endpoints. Treatment continuity should not be equated with survival, and earlier systemic therapy cannot be assumed to improve overall survival in every patient. Likewise, no randomized comparison of PSH and major hepatectomy exists, and selection bias affects all observational comparisons. The proposed concept is therefore deliberately modest: the choice of operation influences which treatments remain technically and physiologically available later, and that consequence deserves to be measured alongside the margin achieved at the index hepatectomy.

Proposing these measures is not the same as making them measurable, and the objection that treatment continuity is at present a qualitative construct is well founded. Table 2 therefore sets out, alongside the reporting elements needed to characterize margin phenotype, an explicit operational definition for each of the four measures: numerator, denominator, index date and principal source of bias. Three of those choices carry most of the weight: independent adjudication of PHLF, separation of indication from capacity in time to systemic therapy, and competing-risks analysis of subsequent curative-intent local treatment.

The analytical hazards are equally specific. Repeat hepatectomy and ablation can only occur after the index operation, so any comparison that counts them from the date of hepatectomy without accounting for survival time will favor whichever group lives longer; this immortal-time problem requires time-dependent exposures or landmark analysis. Selection by indication cannot be handled by adjustment alone, so a registry should record the anatomical scenario prospectively—the number and distribution of lesions, the structures contacted, and whether a detachable plane was identified on imaging and at operation—and emulate a target trial within narrowly defined scenarios rather than comparing all parenchymal-sparing with all major resections. Adjustment should at minimum include tumor number and distribution, extrahepatic disease, RAS and BRAF status, response to preoperative chemotherapy, performance status, and cumulative preoperative chemotherapy exposure. The clinically meaningful magnitude of difference for each proposed measure has not yet been established and should be prespecified in a dedicated prospective protocol rather than inferred from retrospective data.

## 6. Toward an Individualized Framework for Operative Planning

### 6.1. Four Axes of Assessment

**Figure 6:**
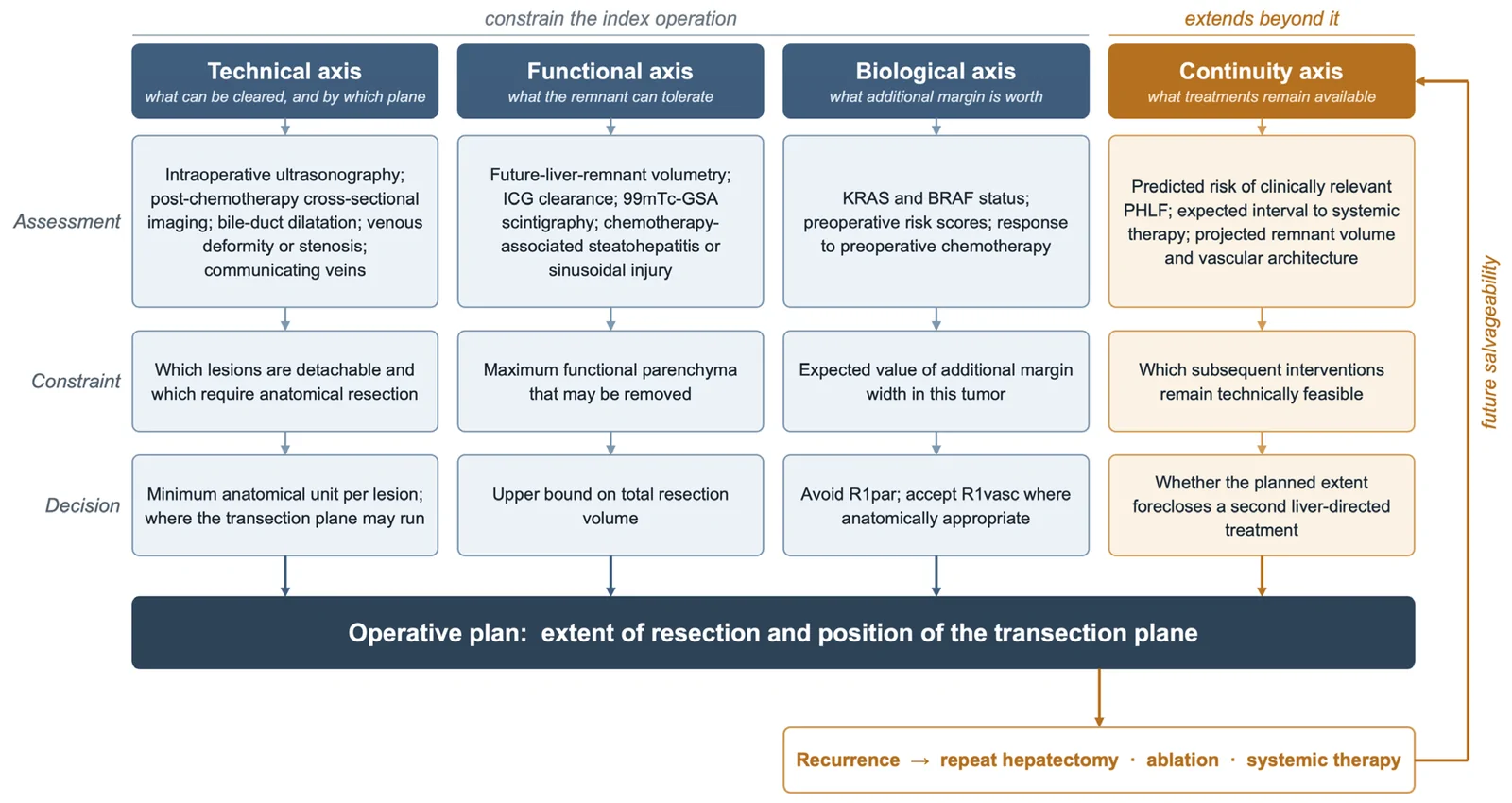
A four-axis framework for operative planning in multiple bilobar colorectal liver metastases. The technical axis determines what can be cleared and by which anatomical plane; the functional axis sets the limit on parenchymal expenditure; the biological axis modifies how much additional margin is likely to be worth; and the continuity axis asks which subsequent treatments will remain available. The first three axes constrain the index operation, whereas the fourth extends beyond it, closing the loop from recurrence back to repeat hepatectomy, ablation and systemic therapy. The framework is a clinical checklist, not a validated score; the axes have not been weighted against one another in prospective data.

The preceding evidence can be organized into four axes that are already considered in routine practice but rarely integrated explicitly: the technical axis (what can be cleared and by which anatomical plane), the functional axis (what the remnant can tolerate), the biological axis (how likely local control at the index operation is to translate into durable benefit), and the continuity axis (what treatments remain available afterwards). These axes are summarized in Figure 6 and are proposed as a clinical checklist rather than a scoring system. Their value lies primarily in making the trade-offs explicit when the anatomically simplest operation is not the one that best preserves the subsequent treatment trajectory.

The technical axis is lesion-specific. Intraoperative ultrasonography and current cross-sectional imaging determine whether a contacted vessel can be preserved, whether Glissonean invasion requires anatomical resection, and whether venous sacrifice is possible without reconstruction [16,33,34,35,36]. The functional axis sets the ceiling on parenchymal expenditure. Future-liver-remnant volumetry remains fundamental [43], but chemotherapy-associated steatohepatitis and sinusoidal injury [41,42] mean that quantitative volume should be interpreted together with the quality and, where appropriate, functional assessment of the remnant [44].

The biological axis modifies the expected value of additional margin. Current evidence does not support a validated genotype-specific margin threshold, but KRAS status and other adverse biological features can influence the relationship between margin phenotype and local recurrence [15,24,27]. Poor biology does not automatically justify a larger resection: when the dominant risk is early intrahepatic or extrahepatic relapse, additional parenchymal sacrifice may not address the principal source of failure. The operative priority remains complete macroscopic clearance while avoiding R1par and preserving major vascular structures when detachment is anatomically appropriate.

Four statements make this axis usable without overstating the evidence. First, molecular status should not be used to set a margin in millimetres. No prospective data support a genotype-specific threshold, and the retrospective signal favoring a 5 mm margin in KRAS-mutant disease [24] has not been replicated as a decision rule. Second, adverse biology may justify a higher threshold for sacrificing functional parenchyma, because in such patients the dominant mode of failure is typically early recurrence elsewhere in the liver or outside it, which a wider transection plane does not prevent, while the parenchyma spent in obtaining that plane is unrecoverable when recurrence requires further treatment. Third, adverse biology should prompt greater caution regarding R1par specifically: the survival detriment associated with parenchymal exposure was largest in KRAS-mutant tumors in the available multicenter data [15], so where a plane is uncertain the appropriate response is a wider parenchymal margin at that lesion, or anatomical resection of its territory, rather than acceptance of exposure. Fourth, available retrospective data have not demonstrated a genotype-specific excess risk associated with R1vasc, but this absence of a signal does not establish oncological safety [15]. Genotype alone should therefore not currently mandate conversion of an anatomically appropriate planned detachment into venous resection or major hepatectomy. In practice, biology more often changes the sequence of treatment than the extent of the operation: when a tumor–vessel interface remains borderline in high-burden RAS- or BRAF-mutant disease, one reasonable approach may be further systemic therapy followed by re-imaging to determine whether the interface becomes clearly detachable [36], when oncologically appropriate, rather than committing immediately to a larger index resection.

The continuity axis asks what the planned operation leaves possible. Before committing to a major resection, the surgeon can consider whether the projected remnant is likely to recover without clinically relevant PHLF, whether planned systemic therapy can reasonably resume, whether a second resection would remain technically feasible in the projected remnant, and whether future percutaneous or intraoperative ablation would remain possible [7,17,48]. These questions do not have numerical answers, but making them explicit turns “future salvageability” from an assumption into a component of operative planning.

### 6.2. Applying the Framework Without Converting It into a Score

The framework is most useful when the axes conflict. If a vessel can be safely detached and complete macroscopic clearance achieved with minimal parenchymal loss, preservation is favored. If imaging or intraoperative findings indicate true invasion of a major pedicle or non-detachable vein, the technical axis overrides the continuity preference and anatomical or vascular resection is required. If one-stage clearance remains unsafe despite maximal parenchymal preservation, staged strategies retain their role [29,30,31]. The framework therefore does not privilege PSH at all costs; it asks that the anatomical price of a wider margin be weighed against its incremental oncological value.

Two of the cases illustrated above show how the axes are weighed in a multidisciplinary discussion. In the patient of Figure 2, with more than 40 bilobar metastases, the technical axis admitted two plans that both achieved complete macroscopic clearance and so did not settle the operation. The functional axis did: right hemihepatectomy projected a remnant of 331 mL (27% of total liver volume) and a functional remnant of 22%, against 521 mL (43%) and 40% for extended posterior sectionectomy combined with multiple partial resections, in a liver that had already received preoperative chemotherapy. The biological axis offered nothing in favor of the larger resection, since the burden of disease made early recurrence the dominant risk and no additional margin at any lesion was gained by removing the right hemiliver. The continuity axis pointed the same way: a 27% remnant leaves little capacity for a second hepatectomy, whereas a 43% remnant preserves it. Against this stood the one real cost of the parenchymal-sparing plan—a cut surface increased from 140 to 296 cm^2^, with the attendant risk of bile leak and hemorrhage—a cost borne by the index operation and accepted because three of the four axes favored preservation.

The patient of Figure 5, with more than 50 metastases, shows the opposite resolution. Here, the continuity axis argued strongly for preserving all three hepatic veins, and the operation did ultimately preserve 79% of the liver volume. The left hepatic vein, however, was involved over two-thirds of its circumference, which the technical axis treats as invasion rather than contact, and no detachable plane existed. The axis that constrains the operation therefore overrode the axis that would have preserved the most tissue: that vein was resected and reconstructed with an autologous inferior mesenteric vein patch, while detachment was used only at the right hepatic vein, where a plane was demonstrable. In both discussions the framework generated no score. Its function was to force the question that is easiest to leave implicit—what will still be possible at the next recurrence—to be asked while the transection plane was still being chosen.

We deliberately do not assign numerical weights to the four axes. No prospective dataset has quantified their relative importance, and the continuity axis is supported mainly by associations rather than demonstrated causation. A score would therefore create spurious precision. The purpose of the framework is simpler: to ensure that the expected postoperative treatment trajectory is considered when the transection plane is chosen, rather than only after recurrence makes the consequences irreversible.

## 7. Limitations and Future Directions

The evidence supporting margin-aware PSH is predominantly retrospective. Patients selected for PSH differ systematically from those undergoing major hepatectomy, definitions of R1vasc vary between centers, and operative intent cannot always be reconstructed reliably from pathology reports. The VESPAH series is particularly limited by its small single-center cohort, long treatment period, unmatched comparison groups, and lack of a demonstrated survival advantage [16]. The recent meta-analysis showing increased local recurrence after R1vasc in minimally invasive CRLM series further underscores the need for careful anatomical selection and technical standardization across centers [14].

Standardized reporting is therefore a priority. Margin phenotype should be recorded per lesion or resection area rather than only per patient, because a patient with several R0 areas and one planned vascular detachment is biologically and technically different from a patient with diffuse parenchymal exposure. R1 should be prospectively subclassified as parenchymal or vascular, with the involved vessel and operative intent documented. When detachment is performed, the presence of an identifiable anatomical plane and the specific structure preserved should also be recorded. The elements we propose as a minimum dataset are listed in Panel (A) of Table 2, and studies of surgical strategy should additionally report the components of treatment continuity defined in Panel (B). These changes would allow future observational analyses to distinguish local margin biology from the downstream consequences of parenchymal preservation.

Prospective multicenter registries are a realistic next step. They should capture margin phenotype, liver-function recovery, time to systemic therapy, recurrence pattern, and every subsequent liver-directed intervention, using the definitions and analytical safeguards set out in Section 5.5 and Table 2. Biomarkers such as postoperative circulating tumor DNA may eventually help identify patients in whom the ability to deliver further therapy is especially important; postoperative minimal residual disease is strongly prognostic after CRLM resection, but biomarker-guided surgical planning remains hypothesis-generating [52]. Rather than using biomarkers to justify wider or narrower margins directly, future studies should test whether biological risk modifies the value of preserving treatment options.

Such registries should also separate local and systemic failure. The relevant endpoint for a planned vascular detachment is recurrence at the specific detachment site, whereas the relevant endpoint for treatment continuity is the proportion of patients who remain eligible for further curative-intent treatment after any hepatic recurrence. Combining these outcomes into a single recurrence-free-survival endpoint obscures the distinction between local technical failure and progression driven by tumor biology. Time-dependent analyses will be particularly important because repeat hepatectomy and ablation occur after the index operation and are strongly influenced by survival and selection up to the time of recurrence.

Randomized comparison of PSH and major hepatectomy is unlikely to be feasible for the full spectrum of CRLM because anatomy often dictates the operation. More focused prospective comparisons may be possible—for example, planned detachment versus venous resection for carefully defined hepatic-vein contact without deformity, using detachment-site recurrence as a local endpoint and remnant function and re-treatability as complementary endpoints. Prospective registries could also support target-trial emulation comparing strategies within narrowly defined anatomical scenarios. Until such data emerge, the appropriate conclusion is not that R1vasc is equivalent to R0 or that PSH improves survival, but that margin phenotype, parenchymal preservation, and longitudinal treatment capacity should be reported and evaluated together.

## 8. Conclusions

R0 resection remains the preferred objective of surgery for CRLM, and nothing in this review supports indiscriminate acceptance of tumor exposure at a parenchymal transection surface. The contemporary margin question is more nuanced: R1par and planned R1vasc represent different anatomical events, and the oncological meaning of either depends on case selection and tumor biology.

For patients with deep, vessel-adjacent or multiple bilobar metastases, the operation should therefore be designed around two simultaneous goals—complete macroscopic clearance and preservation of sufficient functional, vascularized liver for the treatments that may follow. Vessel skeletonization and carefully selected tumor–vessel detachment are technical means to that end, not goals in themselves. Thermal ablation further extends the same parenchymal-preserving principle when an adequate ablative margin can be achieved.

We propose that treatment continuity—preservation of the physiological and anatomical capacity to receive indicated systemic therapy and repeat curative-intent local treatment—be reported alongside margin status, morbidity, recurrence, and survival. In a disease frequently managed through successive interventions, the liver and vascular architecture sacrificed at the first hepatectomy are not only the price of the present margin; they also constrain the options available at the next recurrence. The quality of CRLM surgery should therefore be judged by both the clearance achieved today and the treatment possibilities preserved for tomorrow.

## Figures and Tables

**Table 1:** Parenchymal preservation and the downstream treatment course: what is and is not reported.

Study [Ref]	Design and Population	Local Control/Margin	Survival	Subsequent Treatment
Mise 2016 [7]	Single-center retrospective comparative cohort; solitary CRLM ≤ 30 mm; PSH 156 vs. non-PSH 144	No significant difference in liver-only recurrence (14% vs. 17%) or liver recurrence-free survival.	No significant OS difference in the overall cohort. Among patients with liver-only recurrence, 5-year OS was 72.4% vs. 47.2% (*p* = 0.047) in an unadjusted subgroup analysis.	Among patients with liver-only recurrence, repeat hepatectomy was performed in 15/22 (68%) after PSH and 6/25 (24%) after non-PSH.
Matsumura 2016 [8]	Single-center retrospective cohort; ≥4 CRLM, each ≤50 mm; PSH 113 vs. MH 32	Positive margins: 8.8% vs. 9.4%; liver-only recurrence: 43.4% vs. 50.0%.	5-year OS: 37.0% (PSH) vs. 29.4% (MH) (*p* = 0.473).	Repeat hepatectomy was performed in 53/65 patients (81.5%) with liver-only recurrence across the combined PSH and MH cohort.
Torzilli 2009 [6]	Prospective single-arm series; one-stage ultrasonography-guided resection of multiple bilobar CRLM	Complete macroscopic clearance was achieved using a one-stage parenchymal-sparing approach.	Single-arm study; no comparative survival estimate.	Demonstrated feasibility of one-stage clearance; the single-arm design does not provide a controlled estimate of subsequent treatment capacity.
Torzilli 2019 [31]	Multicenter case-matched cohort; e-OSH vs. ALPPS	R0 (≥1 mm): 21.2% (e-OSH) vs. 80.8% (ALPPS). The lower R0 rate after e-OSH reflects planned tumor–vessel detachment, performed in 23/52 e-OSH patients (44.2%). R1 subtypes were not reported for ALPPS.	Median OS: 32.6 (e-OSH) vs. 31.7 months (ALPPS); no significant difference in OS or DFS.	After liver-only recurrence, further liver-directed treatment was performed in 10/16 (62.5%) after e-OSH and 3/7 (42.9%) after ALPPS; this was not a main endpoint.
Wang 2023 [9]	Systematic review and meta-analysis; PSR vs. AR; 22 studies, 7228 patients	Positive-margin and intrahepatic-recurrence rates were higher after PSR; liver recurrence-free survival did not differ significantly.	No significant difference in OS or DFS.	Repeat hepatectomy was more frequent after PSR in a pooled analysis of 14 studies. This outcome does not directly measure treatment eligibility among patients with recurrence.
Umeda 2022 [16]	Single-center retrospective cohort; ≥8 bilobar CRLM; VESPAH 20 vs. MH 13	No significant difference in recurrence rate or pattern.	Median RFS: 8 vs. 8 months (VESPAH vs. MH); OS did not differ significantly.	PHLF grade B/C: 1/20 (5%) vs. 5/13 (38%). Chemotherapy within 2 months: 18/20 (90%) vs. 5/13 (38%). Repeat hepatectomy after intrahepatic recurrence: 12/14 (86%) vs. 4/7 (57%).

Values follow the comparison order in the design column unless stated otherwise. CRLM, colorectal liver metastases; PSH, parenchymal-sparing hepatectomy; PSR, parenchymal-sparing resection; MH, major hepatectomy; AR, anatomic resection; e-OSH, enhanced one-stage hepatectomy; ALPPS, associating liver partition and portal vein ligation for staged hepatectomy; OS, overall survival; RFS, recurrence-free survival; DFS, disease-free survival; PHLF, posthepatectomy liver failure; VESPAH, vessel-skeletonized parenchymal-sparing hepatectomy. All comparative studies listed are retrospective and non-randomized. Repeat-treatment denominators and definitions differ across studies, and the meta-analysis overlaps with the individual studies listed.

**Table 2:** Proposed reporting standards: (**A**) a minimum set of elements for characterizing margin phenotype and (**B**) operational definitions for the four candidate measures of treatment continuity.

(A) Minimum Reporting Elements for Margin Phenotype				
Element	What to Record			Why It Matters
Unit of analysis	Margin status per lesion and per resection area, in addition to per patient			Patient-level R0/R1 conflates several distinct margins when one operation contains many resection areas.
Margin definition	Whether R1 denotes tumor at the inked surface or a clearance of less than 1 mm, stated explicitly			The same cohort yields materially different R1 rates under the two definitions.
Margin width	Measured clearance in millimetres for each resection area			Permits re-analysis under either definition and pooling across studies.
Margin phenotype	R0, R1par or R1vasc for each resection area			Separates an unplanned transection-surface event from a planned detachment.
Involved structure	For R1vasc: hepatic vein trunk, named venous tributary, or Glissonean pedicle with its order			Venous and Glissonean detachment are not equivalent; pedicle involvement may extend along biliary and arterial components.
Operative intent	Detachment planned preoperatively, decided intraoperatively, or unintended exposure			Distinguishes a strategy from an accident; the two should not be pooled.
Detachable plane	Whether an intact plane was identified on preoperative imaging, at intraoperative ultrasonography, and at dissection			This plane is the anatomical premise on which R1vasc rests.
Contraindicating findings	Arc of contact, vessel deformity or stenosis, circumferential encasement, upstream bile-duct dilatation			Records the findings that should preclude detachment, allowing selection to be audited.
Site-specific recurrence	Recurrence at each detachment site, reported separately from other intrahepatic recurrence			The only endpoint that tests detachment itself rather than the biology of the disease.
**(B) Operational definitions for the candidate measures of treatment continuity**				
**Measure**	**Numerator**	**Denominator**	**Index date**	**Analysis and principal bias**
(i) Clinically relevant PHLF	ISGLS grade B or C	All patients undergoing hepatectomy	Day of hepatectomy; 90-day window	Grade B turns on a change in clinical management. Adjudicate prospectively by an assessor who did not perform the operation, and report inter-observer agreement.
(ii) Time to systemic therapy	First administered dose of the planned regimen	Patients in whom systemic therapy was indicated	Day of hepatectomy	Indication is not capacity. Record the intended regimen and the reason for every non-delivery, so that “not indicated” is not pooled with “unable to receive”.
(iii) Curative-intent local re-treatment	Resection, repeat hepatectomy or ablation performed with curative intent	Patients with hepatic recurrence	Date of hepatic recurrence	The denominator is reached only by patients who survive to recur. Report as cumulative incidence with death and isolated extrahepatic progression as competing events.
(iv) Cumulative liver-directed interventions	Number of resections and ablations delivered	All patients undergoing hepatectomy, per person-year of follow-up	Day of hepatectomy	Immortal time: longer survival permits more interventions. Use time-dependent exposures or a landmark analysis.

ISGLS, International Study Group of Liver Surgery; PHLF, posthepatectomy liver failure; R0, negative resection margin; R1par, parenchymal R1 exposure; R1vasc, planned vascular R1 detachment. Panel (**A**) lists the elements proposed as a minimum dataset for characterizing margin phenotype (Section 3.1 and Section 7); Panel (**B**) gives operational definitions for the measures proposed in Section 5.5. Neither panel is a validated instrument; both are offered as a starting point for prospective registries, and each measure requires the analytical safeguards listed before it can be compared between strategies.

## Data Availability

No new data were created in this review. The data supporting the illustrative findings are available in the original publication [16].

## Supplementary Materials

The following supporting information can be downloaded at https://www.mdpi.com/article/10.3390/cancers18213492/s1. Table S1: Key studies informing the contemporary interpretation of the resection margin in colorectal liver metastases. Video S1: Intraoperative demonstration of vessel-skeletonized parenchymal-sparing hepatectomy (the case shown in Figure 5).
