# Supplementary material for: Rethinking Surgical Margins in Colorectal Liver Metastases: Vessel-Skeletonized Parenchymal-Sparing Hepatectomy (VESPAH) and Treatment Continuity
Source: Cancers (Basel). 2026 Oct 29;18(21):3492. doi: 10.3390/cancers18213492 (PMC13762380; doi:10.3390/cancers18213492)
Supplement: Supplementary file 1 [file cancers-18-03492-s001.zip › Supplementary File/VESPAH_Supplementary_TableS1_v2.pdf]

Supplementary Material

Rethinking Surgical Margins in Colorectal Liver Metastases: Vessel-Skeletonized Parenchymal-Sparing Hepatectomy (VESPAH) and Treatment Continuity

Table S1. Key studies informing the contemporary interpretation of the resection margin in colorectal liver metastases.

Study [ref]	Design	Margin categories	Principal finding relevant to this review
Ekberg 1986 [19]	Retrospective cohort	<1 cm vs ≥1 cm	A margin <1 cm was associated with poorer survival, providing historical support for the 1-cm margin rule.
Kokudo 2002 [20]	Histological study of resected specimens	Microscopic extension beyond the gross tumor border	Microscopic extension was generally confined to the immediate peritumoral region, supporting consideration of narrow negative margins; the authors proposed a minimum margin of 2 mm.
Pawlik 2005 [1]	Multi-institutional retrospective cohort	R0 (≥1 mm) vs R1; width categories within R0	Survival did not differ significantly by margin width among R0 resections. A positive margin was associated with more surgical-margin recurrence and worse survival.
Hamady 2014 [2]	Propensity score-matched cohort analysis	≥1 mm vs <1 mm; comparisons with wider margins	Disease-free survival did not improve further with margins wider than 1 mm in propensity score-matched comparisons.
Andreou 2013 [21]	Single-center retrospective cohort after preoperative chemotherapy	R0 vs R1	R1 status remained independently associated with worse overall survival; its adverse association was particularly evident in patients with a poor response to chemotherapy.
Margonis 2018 [3]	Systematic review and meta-analysis	Width comparisons using 1-mm and 1-cm thresholds	Wider negative margins were associated with better survival in threshold-based analyses, particularly at the 1-cm threshold; these comparisons do not establish a continuous increase in benefit with every increment in width.
Östrand 2024 [4]	Population-based national cohort	0.0 mm vs 0.1–0.9 mm vs ≥1 mm	A margin ≥1 mm remained associated with better overall survival after adjustment in a contemporary national cohort that included patients receiving perioperative chemotherapy.
Sadot 2015 [23]	Single-center retrospective analysis of a prospectively maintained database (n = 2,368)	0-mm margins vs submillimeter and wider negative margins	Margin width remained independently associated with overall survival. Even submillimeter clearance was associated with better survival than a 0-mm margin, a finding the authors suggested may also reflect tumor biology.
Iwaki 2023 [24]	Single-center retrospective, genotype-stratified analysis (146 lesions in 67 patients)	R0 vs R1; <5 mm vs ≥5 mm within R0	Within R0 resections, <5-mm margins were associated with more local recurrence in KRAS-mutant tumors, whereas no significant difference was detected in KRAS wild-type tumors. Lesions contacting Glissonean pedicles were excluded.
Viganò 2016 [11]	Observational cohort with per-resection-area analysis	R0 vs R1par vs R1vasc	Local recurrence per resection area was 1.5% after R0, 3.9% after R1vasc, and 13.6% after R1par, supporting separate reporting of parenchymal exposure and vascular detachment.
Torzilli 2018 [12]	Consecutive cohort at the hepatocaval confluence (135 patients; 190 tumor–	Vessel-contact lesions managed by detachment or	A vein- or parenchyma-sparing strategy was feasible in most patients. The cohort included detachment and venous resection/reconstruction; the reported local

	vein contacts)	venous resection	recurrence rate was 6% at a median follow-up of 27 months.
Viganò 2018 [13]	International survey of 276 HPB surgeons from 52 countries	R1par vs R1vasc: perceptions and practice	Acceptance of vascular detachment varied: 45% of respondents considered a vascular margin equivalent to R0, while willingness to use detachment depended on the clinical scenario.
Procopio 2020 [27]	Observational cohort, genotype-stratified	R0 vs R1par vs R1vasc	In KRAS-mutant tumors, local recurrence was more frequent after R1par than after R1vasc. In KRAS wild-type tumors, per-area local recurrence was higher after R1vasc than after R0.
Procopio 2026 [14]	Meta-analysis of minimally invasive liver surgery; CRLM subgroup	R0 vs R1vasc	In the CRLM subgroup, R1vasc was associated with more local recurrence than R0, while no statistically significant difference in overall survival was detected.
Ferrari 2026 [15]	Multicenter cohort with documented KRAS/BRAF status and margin type (n = 1,078)	R0 vs R1par vs R1vasc	R1par was associated with worse overall survival, particularly in KRAS-mutant tumors. No significant survival disadvantage was detected for R1vasc across molecular subgroups; the small BRAF-mutant subgroup (n = 35) limits precision.

CRLM, colorectal liver metastases; HPB, hepatopancreatobiliary; R0, negative resection margin; R1, positive resection margin; R1par, parenchymal R1 margin; R1vasc, vascular R1 margin. Margin definitions and cutoffs follow the source studies and are not uniform across studies. A nonsignificant difference does not establish oncological equivalence. References are numbered as in the main text.

Video S1. Intraoperative demonstration of vessel-skeletonized parenchymal-sparing hepatectomy (the case shown in Figure 5). Adapted from the Supplementary Video accompanying Umeda Y, et al. Langenbecks Arch. Surg. 2022, 407, 685–697 [16], under the Creative Commons Attribution 4.0 International License (http://creativecommons.org/licenses/by/4.0/).
